# The Role of the Plant Antioxidant System in Drought Tolerance

**DOI:** 10.3390/antiox8040094

**Published:** 2019-04-08

**Authors:** Miriam Laxa, Michael Liebthal, Wilena Telman, Kamel Chibani, Karl-Josef Dietz

**Affiliations:** Department of Biochemistry and Physiology of Plants, Faculty of Biology, University of Bielefeld, Universitätsstr. 25, 33615 Bielefeld, North Rhine Westphalia, Germany; mliebthal@uni-bielefeld.de (M.L.); wtelman@uni-bielefeld.de (W.T.); kamel.chibani@uni-bielefeld.de (K.C.); karl-josef.dietz@uni-bielefeld.de (K.-J.D.)

**Keywords:** antioxidant, drought, ROS, RNS, stress, acclimation

## Abstract

Water deficiency compromises plant performance and yield in many habitats and in agriculture. In addition to survival of the acute drought stress period which depends on plant-genotype-specific characteristics, stress intensity and duration, also the speed and efficiency of recovery determine plant performance. Drought-induced deregulation of metabolism enhances generation of reactive oxygen species (ROS) and reactive nitrogen species (RNS) which in turn affect the redox regulatory state of the cell. Strong correlative and analytical evidence assigns a major role in drought tolerance to the redox regulatory and antioxidant system. This review compiles current knowledge on the response and function of superoxide, hydrogen peroxide and nitric oxide under drought stress in various species and drought stress regimes. The meta-analysis of reported changes in transcript and protein amounts, and activities of components of the antioxidant and redox network support the tentative conclusion that drought tolerance is more tightly linked to up-regulated ascorbate-dependent antioxidant activity than to the response of the thiol-redox regulatory network. The significance of the antioxidant system in surviving severe phases of dehydration is further supported by the strong antioxidant system usually encountered in resurrection plants.

## 1. Introduction

During their ontogenesis, plants face a dynamically changing environment defined by abiotic factors (e.g., light/dark, temperature, nutrient and water availability, and toxic compounds such as heavy metals) and biotic interactions (e.g., beneficial and pathogenic microbes, fungi, insects, other herbivores) [1]. Environmental perturbations which significantly disturb metabolism, development and yield, are considered as stress situations and cause stress responses in biological system. Such imposed stress is commonly accompanied by an increase in the production of reactive oxygen species (ROS) and reactive nitrogen species (RNS) that lead to an imbalance between their production and scavenging. Despite their reactive and thus toxic nature, ROS and RNS are also key components of signal transduction pathways that trigger stress responses. Furthermore, ROS and RNS are involved in plant developmental processes [2,3,4] and plant-microbe interactions [5,6]. However, excessive ROS and RNS production must be counteracted by the antioxidant system to prevent damage development and cell death.

**Figure 1:**
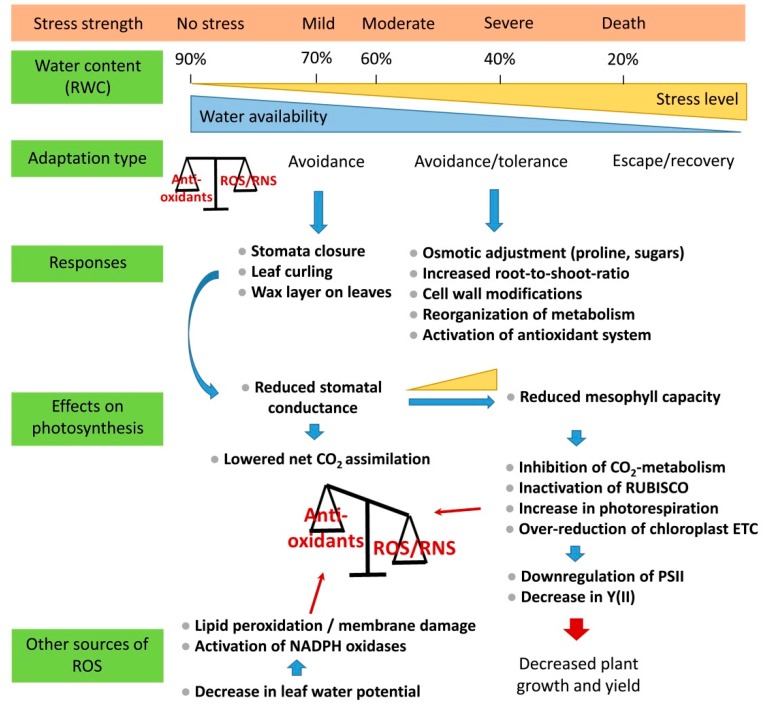
Physiological and biochemical processes triggered by drought.

Drought stress severely impacts plant development, growth and fertility. Drought triggers water loss and a decrease in water potential, which concomitantly leads to a reduction in cell turgor (Figure 1). Among the fastest processes induced by drought is the abscisic acid (ABA)-mediated closure of stomata [7]. Prolonged drought stress and increased stress intensity lead to further acclimation reactions. These responses include osmotic adjustment [8,9], decreased shoot-root ratio [10], cell wall modifications [11,12], reprogramming of metabolism [13], and activation of the antioxidant system [14,15]. Many of these modifications are measurable and are used to characterize the severity of drought stress. Measurable traits are, for example, the stomatal and mesophyll conductance, net photosynthesis, photorespiration, abundance of osmoprotectants, tissue water potential, ABA content and membrane integrity. Drought avoidance includes morphological adaptations, like leaf curling and increased wax deposition on the leaf surface [16] (Figure 1).

**Figure 2:**
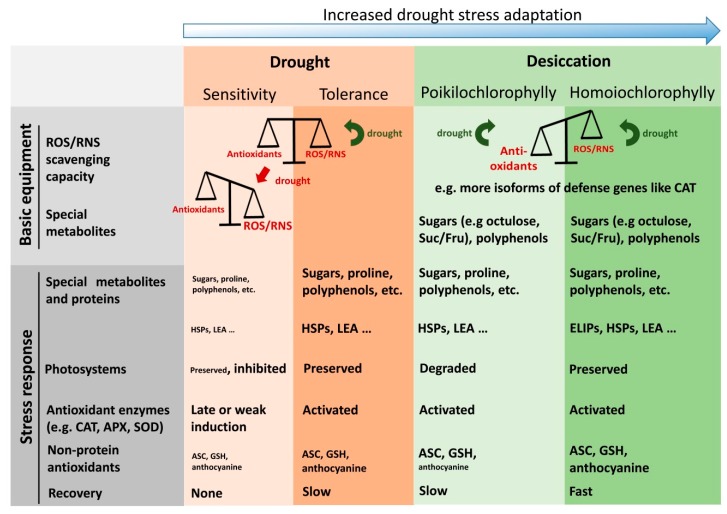
Characteristic features of drought-sensitive, drought-tolerant and desiccation-tolerant plants. The figure summarizes properties related to metabolism, antioxidant defense, and recovery which often are associated with the physiological traits. Red arrow: reactive oxygen species (ROS)/reactive nitrogen species (RNS) gain prevalence; green arrow: status is preserved following drought. Fond size correlates with the strength of stress responses measured. ROS, reactive oxygen species; RNS, reactive nitrogen species; HSP, heat shock protein; LEA, late embryogenesis abundant protein; ELIP, early light-inducible protein; Suc/Fru, sucrose to fructose ratio; CAT, catalase; APX, ascorbate peroxidase; SOD, superoxide dismutase; ASC, ascorbate; GSH, glutathione.

During evolution, plants developed mechanisms to acclimate to drought or even to withstand dry periods. Extensive research has unraveled the molecular mechanisms of drought and desiccation tolerance. Figure 2 summarizes characteristic features of drought-sensitive, drought-tolerant and desiccation-tolerant plants. Tolerant plants are equipped with higher levels of both osmolytes and non-protein antioxidants, reprogram their metabolism and enhance their antioxidant capacity. Interestingly, sensitive species also activate their antioxidant system. Nevertheless, despite this apparent contradiction, drought tolerance seems to be a function of the antioxidant capacity realized in response to drought. Furthermore, the antioxidant activity not only is important during acute drought stress, but also interferes with recovery from water limitation and resurrection from dehydration.

In the beginning of the review we will recall the classification of drought and how drought stress conditions are experimentally induced. This is important information to relate the production of ROS and RNS to the applied stress later in this review. Our review centers on the sites of production and roles of ROS and RNS during dehydration and their detoxification by the antioxidant system. Where possible we will correlate the activation of the antioxidative system to drought tolerance. Furthermore, we will evaluate which antioxidants are involved in drought response in particular. The last section describes the role of the antioxidative system in resurrection plants as an intriguing case of exceptional drought tolerance.

## 2. Classification and Application of Drought Stress

Drought is classified in mild, moderate and severe stages of stress (Table 1). The transition between the different stages occurs steadily and reflects the progression of drought stress severity both in duration and dehydration strength. Hence, an absolute value of dehydration cannot be assigned to the individual stages of drought stress. The stages are rather categorized in certain ranges. Various units have been used to describe water limitations (Table 1). The overall consensus is that the relative water content (RWC) in mild drought stress ranges between 60–70% compared to the control of ≥90%, in moderate stress between 40–60% and in severe stress between 0–40% (Table 1, Figure 1). Interestingly, these classifications are quite consistent between different species, even though the length of the applied stress to reach these states differs considerably (Table 1). Severe drought stress conditions can be reached rapidly within a week in soils with low water holding capacity. Mild stress conditions, corresponding to a soil field capacity (SFC) of 70%, are already reached after two days, severe (SFC < 50%) and very severe wilting (SFC < 30%) after five and eight days, respectively, as determined for 25 day-old soybeans grown in a sand-vermiculite mixture [17]. A time period of 1–2 weeks without watering was shown to be the most suitable condition for testing both drought tolerance and recovery of various mesophytic species grown on soil (Table 2). Drought stress can be induced either by withholding water in the case of soil-grown plants or by polyethylene glycol (PEG) in both agar-plates and liquid cultures [18]. The use of PEG-infused agar systems allows generating a defined water potential in the substrate [19]. However, the majority of these systems were only applicable for seedlings for a long time. Recently, Frolov and colleagues [20] established an agar-based polyethylene glycol infusion drought model for six-to-eight-week-old *Arabidopsis* plants. This system is extremely valuable as it allows analyzing the response of adult plants and thus a more appropriate developmental stage in terms of agricultural application.

The occurrence and severity of drought-induced injury varies between different developmental stages of the plant and also depends on duration and strength of the applied stress.

## 3. ROS and RNS Generation during Dehydration and Its Combination with Other Stresses

Stress-induced production of ROS and RNS occurs in different cell compartments [45]. They are used to transmit signals to the nucleus and other compartments to reprogram cell performance including gene expression [46,47]. The underlying mechanisms are known as retrograde and anterograde signaling pathways [1,48]. This paragraph focuses on the sources of ROS and RNS, and their accumulation in response to drought stress.

### 3.1. ROS during Drought

The first response of plants to drought is the closure of stomata in order to minimize water loss due to transpiration. Because of ongoing photosynthesis in the light, the increased gas diffusion barrier facilitates depletion of the intercellular carbon dioxide (CO_2_) concentration. Decreased availability of CO_2_ stimulates ribulose–1,5–bisphosphate oxygenation and, thus, photorespiratory hydrogen peroxide (H_2_O_2_) production in the peroxisomes. This effect has been studied in detail and was frequently summarized, e.g., with respect to drought and H_2_O_2_ production in wheat and potato as C_3_ field crops [49]. Insufficient availability of the electron acceptor CO_2_ slows down the oxidation of nicotinamide adenine dinucleotide phosphate (NADPH) in the Calvin–Benson cycle. Lack of NADP^+^ causes a backlog of electrons and over-reduction of the photosynthetic electron transport which in turn increases the reduction rate of oxygen as alternative electron acceptor in the Mehler reaction at photosystem I (PSI) and enhanced release of superoxide anion (O_2_●^−^) and hydrogen peroxide (H_2_O_2_). Hence, chloroplasts are primary targets of excess light and CO_2_ starvation in drought. In addition, photorespiration produces NADH in the mitochondrion.

A highly reduced chloroplast NADPH-pool via thioredoxin (TRX) reduction activates the NADPH-dependent malate dehydrogenase and, thereby, the malate valve for export of reducing equivalents to the cytosol and mitochondrion. The disequilibrium between electron supply and consumption in photosynthesis is efficiently transmitted to the respiratory electron transport chain (ETC) in the mitochondrion. Activation of alternative oxidase (AOX) and induction of *aox* gene expression are hallmarks of drought response [50,51,52]. Even under normal conditions, 1–2% of oxygen is consumed to produce ROS due to an over-reduction at complex I and III in the oxidative phosphorylation [53]. Under drought, the capacities of AOX, plant uncoupling proteins (PUCPs) and ATP-sensitive potassium channels are stimulated to dissipate excess electron flow in ETC [54]. Respiratory functions are inhibited by about two-thirds in drought-stressed plants as reviewed by Atkin and Macherel [55]. These studies included dehydration regimes of various intensities and on different time scales. The authors commented that the missing response in tolerant species might be due to enhanced antioxidant defense. Additionally, ROS are produced at the apoplast. Interestingly, the production of apoplastic ROS is coupled to calcium signaling [56]. Respiratory burst oxidase homolog (RBOH) proteins in the plasma membrane are calcium and phosphorylation-sensitive enzymes generating superoxide anions in the apoplast in response to drought, but also many other stresses [57,58]. Cell wall-associated kinases (WAKs) are members of the receptor-like kinase (RLK) family and participate in the perception of turgor pressure changes during drought probably linking ROS bursts with phosphorylation of RBOHs [59]. Apoplastic ROS also induce lipid peroxidation giving rise to malondialdehyde (MDA) as an indicator for membrane damage especially during drought. After dismutation of superoxide to H_2_O_2_ in the apoplast, transfer of H_2_O_2_ from the apoplast to the cytosol may also contribute to the intracellular ROS signature.

Table 3 summarizes changes of ROS and RNS amounts in response to drought stress. Maize growing in soil at 20% water saturation deficit accumulated twice the H_2_O_2_ amount of well-watered control plants [60]. Likewise, H_2_O_2_ reached thrice the contents of control rice if exposed to 200 mmol/L mannitol for two days [61,62] and in *Ailanthus altissima* plants that were kept unirrigated for 14 days [63], respectively. Thus, accumulation of ROS under drought is a prototypic case of stress-induced responses.

### 3.2. ROS, Oxidative Post-Translational Modifications and Redox Signalling

Within proteins, the thiol groups of both cysteine (Cys) and methionine (Met) are the major sites of oxidative post-translational modifications (PTMs) [90]. Thiols are prone to successive oxidation to sulfenic (R-SOH), sulfinic (R-SO_2_H), and sulfonic (R-SO_3_H) acids [91]. Cys oxidation and reduction efficiently regulates enzyme activities. A well-established system is the redox system of chloroplasts in which the redox input is provided by ferredoxin (Fd), NADPH and glutathione (GSH), redox signals are transmitted on target proteins by TRX, NADPH-thioredoxin reductase (NTRC) and glutaredoxins (GRX) [92]. Peroxiredoxins (PRX) are thought to sense the redox state of the cell and act in signaling instead of ROS detoxification [92]. Oxidative PTMs and the role of PRX in plant redox signaling are subjects of recent reviews and, thus, are not discussed in detail here [92,93].

### 3.3. RNS during Drought

Reactive nitrogen species are less diverse than ROS. Nitric oxide (NO) is a gaseous signaling molecule involved in germination, development, hormone regulation, and stress management. While homologues of animal NO synthase are absent from plants [94], the described mechanisms for NO production include (i) nitrate reductase (enzymatic, cytosol/plasma membrane), (ii) xanthine oxidoreductase (enzymatic, peroxisome), (iii) NO-associated proteins (enzymatic, mitochondria/plastids), (iv) nitrite: NO reductase (enzymatic, plasma membrane), (v) electron transport chain (non-enzymatic, mitochondria/chloroplast), and (vi) a poorly understood mechanism using arginine, polyamine or hydroxylamine [95,96,97]. The bioactive NO concentration is influenced by the nitrogen nutrient supply, the concentration of the storage compound nitrosoglutathione (GSNO), the activity of the GSNO reductase, and turnover mechanisms including the interaction with hemoglobins [98,99,100].

Osmotic stress, established by exposing rice roots to 200 mmol/L mannitol, increased the NO amount threefold within 24 h in rice leaves [61]. The same increase in NO was observed in rice after withholding irrigation for nine days, while a significant increase was undetected after three days [86]. Since both studies focused on leaves, the large time scale difference is striking and may reflect the time span needed to establish similar stress levels. This interpretation is supported by the fact that an osmotic shock treatment with 210 mmol/L mannitol corresponds to an applied osmotic potential of approximately −1.1 MPa [101], while an equivalent osmotic potential after withholding water was reached only at days 4 and 5 [86]. The data also point to changes in drought sensitivity during development. Most plants respond more sensitive to dehydration in early developmental stages. Therefore, one explanation for the discrepancies between the above mentioned studies might be attributed to differences in the plant growth stages of 16 [61] versus 42 days [86], leaving juvenile leaves more sensitive to drought. In this context, it should be mentioned that the ratio of developing to mature cell in the leaf lamina changes significantly during the early phase of development. Furthermore, the antioxidant response to paraquat was compromised in young *Arabidopsis* leaves [102]. Mature leaves were able to compensate ROS accumulation much more efficiently due to an increase in APX activity. The authors suggested different photoprotective regulatory mechanisms in the two leaf types. Furthermore, it was concluded that the redox-state of plastoquione A (Q_A_) is the determinant of tolerance to paraquat-induced oxidative stress [102]. A similar observation was made in *Fagus sylvatica* L. Here, resistance to paraquat-induced oxidative stress was mediated by an increase in SOD activity in mature leaves [103]. In the tea plant (*Camellia sinensis*), cold-sensitivity of young leaves is correlated with inhibited expression of genes related to cell membranes, carotenoid metabolism, photosynthesis and the antioxidative system [104]. In contrast, transcripts belonging to the gene ontology groups of chloroplasts, cell membranes, redox processes, glutathione metabolism and photosynthesis were increased in mature leaves in response to cold. Hence, the antioxidative system plays an important role in establishing acclimation and hardening to stress.

In tree species like *Ailanthus altissima,* NO amounts increased three-fold after withholding water for 14 days [63]. NO is reported as an important positive regulator for Crassulacean acid metabolism (CAM) in pineapple leaves as described by Freschi et al. [79]. Emission of NO gradually increased from 40 to 140 pmol^.^h^−1^g^−1^ dry weight upon treatment with 30% PEG 6000 for 5 days. Of PEG, 30% corresponds to a water potential of −1.03 MPa [105] and, thus, is similar to osmotic stress induced by 200 mmol/L mannitol [87]. NO quantification was mostly achieved by using fluorescence probes like diaminofluorescein (DAF) or diaminorhodamine (DAR) derivates. To overcome drawbacks related to limited specificity, new probes are presently engineered to improve sensitivity and specificity [106]. Nevertheless, cell- and tissue-imaging with DAF-2 diacetate in dehydrating pineapples localized NO in chlorenchyma, trichoma and epithelial cells but did not resolve subcellular compartmentation.

NO also plays a significant role in regulating germination during drought in grasses like wheat and rice [87,107]. Endogenous NO counteracts programmed cell death and vacuolization induced by gibberellic acid. The NO amount in aleurone layers drops by 75% after 24 h of osmotic stress compared to controls (20% PEG-6000). Exogenous application of NO donors alleviates the effect and delays germination. Thus, a synergistic effect of NO is seen with ABA allowing postponing germination until growth conditions improve. Under such conditions, germination is inhibited and resumed only after growth conditions have improved. Expression of rat neuronal NO synthase (nNOS) in plants constitutively increases NO levels twofold in *A. thaliana* [80] and 1.5-fold in *O. sativa* [61]. These nNOS-plants accumulate more biomass and less H_2_O_2_ after withholding water for 14 d (*A. thaliana*) or upon treating rice with 200 mmol/L mannitol. These results assign a significant role to NO in shaping the acclimation to drought. They also show that the NO effect partly antagonizes the effects of ROS in this process.

In general, information on plant specific endogenous RNS signaling is still scarce. The production of NO occurs in similar subcellular compartments as ROS but our knowledge on its induction, regulation of enzyme activities, and substrates emerges only slowly. Hence, many groups use NO donors to artificially expose plants to RNS. Currently, research focuses on synergistic versus antagonistic effects of RNS and ROS, especially in the field of abiotic stress, and promises a more integrative concept. Experiments on genetic model systems are needed which link the dynamics of specific markers for RNS signaling with proteomic and transcriptomic analyses.

### 3.4. Nitrosylation by ONOO^−^ and GSNO

Antagonistic and synergistic effects relate to reaction products of RNS and ROS and antioxidants, respectively. Thus, GSNO forms by reaction of NO with reduced glutathione, while peroxynitrite (ONOO^−^) forms at sites of simultaneous formation of O_2_●^−^ and NO. GSNO triggers S-nitrosylation, while ONOO^−^ causes tyrosine nitration. Several targets of these reactions are part of the antioxidant defense system like PRX, ascorbate peroxidase (APX), monodehydroascorbate reductase (MDHAR), dehydroascorbate reductase (DHAR) and catalase (CAT) [108,109]. Especially during drought in *Lotus japonicus* NO amounts doubled in roots, but interestingly not in leaves [84]. S-nitrosylation of proteins is promoted in roots. The authors hypothesized that roots are prone to nitrosative stress, and leaves to oxidative stress.

Higher NO concentrations in roots compared to leaves were also reported in sugarcane [89] and bluegrass [110] and support this rule of thumb. One function of NO in roots concerns root patterning as described for pea, tomato, tobacco, and cucumber facing drought conditions [82,111,112,113]. Such differential effects have also been reported for pollen development and stigma function which respond preferentially to either RNS or ROS, respectively. Apparently, ROS and RNS play unique roles in developmental signaling which should be explored further [114]. Furthermore, GSNO serves as a mobile carrier of NO allowing for long distance signaling. In contrast, ONOO^−^ is highly reactive and characterized by a short half-life of 10 to 20 ms, and thus is discussed as a linker between ROS and RNS signaling [115]. Moreover, specific analyses are needed to clarify the NO-related effects on metabolism and to see whether RNS signaling is exclusively transmitted by ONOO^−^ and GSNO.

### 3.5. ROS/RNS in Stress Combinations with Drought

Responses to drought are accentuated if dehydration is combined with other abiotic stresses. Exceptions from this rule concern drought combined with ozone and high CO_2_. The antagonising effect is traced back to stomata closure triggered by ozone [116] or high CO_2_ [117]. Iyer and collegues [116] described this phenomenon in *Medicago truncatula*. Here, ROS levels increase in response to drought and ozone by 2-fold and 2.8-fold, respectively, compared to the well-watered condition. However, ROS levels in response to combined drought and ozone stress are indistinguishable from the control (well-watered plants). In contrast, NO levels are elevated only in response to drought by approximately 2-fold, while ozone has no effect. Simultaneous application of the two stresses again did not lead to significant changes. Interestingly, jasmonic acid and salicylic acid synthesis are induced after application of NO-donors in *A. thaliana* which might explain the mitigating effect of ozone in combination with drought [118]. Again, both reports vary in species and treatment, but indicate that RNS signaling is directly involved in stress response and alters the ROS effects.

In the natural environment, dry periods often coincide with high temperature and high light. Malondialdehyde (MDA) is an indicator for lipid peroxidation and oxidative damage and significantly increases in green tissue of citrus cultivars exposed to a combination of drought and heat (10 d, 40 °C). The increase is absent in single stress applications [119]. The stronger effect of a drought/heat combination is also seen in maize. Here, MDA levels increase by 225%, while the single applications elevated MDA levels by only 45% (−0.7 MPa PEG for 8 h) or 92% (2 °C/h increase from 28 to 42 °C for 8 h), respectively [120]. In cotton cultivars, no significant differences in H_2_O_2_ levels are observed for drought and combined drought/heat stress [121].

Combining heat (42 °C) and drought in succulent purslane for seven days doubles MDA content, while single stress treatments increase the MDA amount only by 20%. Interestingly, O_2_●^−^ amount raises 2.5-fold under heat and combined stress, but not in plants exposed to drought [122]. Surprisingly, the leaf H_2_O_2_ level decreases in grapevine upon deprivation from water for four days followed by treatment with heat (1 h, 42 °C) or high light (1 h, 2000 µmol quanta^.^s^−1^m^−2^) [123]. None of the double or triple stress treatments including drought alters the H_2_O_2_ amounts above the levels measured during control treatments. Significant variations between cultivars are only seen in single treatments and a heat/high light treatment.

These examples support the theory by Suzuki and colleagues [1] that the response to a combined stress is unique and cannot be simply extrapolated from the responses to single stresses. For instance, the response to stress combinations on signaling pathways and responses can be synergistic, antagonistic or independent. Antagonistic and, thus, positive interactions are observed for the combination of drought and high CO_2_ [124]. However, combined stress often leads to negative interactions, and the consequences are synergistic rather than additive [1]. This is also true for high light and drought [125]. Both, high light and drought realize an over-reduced state of photosynthetic ETC. With respect to high light the over-reduction is caused by an excess of light energy, while the over-reduction following drought is caused by a limited CO_2_ availability after stomatal closure and the concomitant inhibition of the Calvin–Benson cycle. Consequently, in both cases ROS and RNS are generated, but the ROS/RNS signatures differ in both cases [126].

The described examples demonstrate the importance to investigate plant responses and signaling pathways in combined stress. However, most laboratory studies on plant stress responses consider one stress at a time, whereas plants in the field usually are exposed to different stresses simultaneously. For example, drought stress is often accompanied by heat and high light intensities [117,127]. Therefore, it has to be kept in mind that any treatment applied under controlled growth chamber conditions fails to reflect field conditions. Ecotypes of the same plant species adopt distinct adaptive responses to acclimate to their local habitats. Such naturally occurring biodiversity in terms of sensitivity vs. tolerance of closely related species, the extreme adaptability of specialists and the special case of crop plant monocultures cannot be treated in this review focusing on ROS and RNS-dependent signaling.

## 4. Response of the Redox Network under Drought

The activation of the antioxidant system via retrograde signaling is a key process in plant acclimation to oxidative stress. Thus, the upregulation of antioxidant enzymes represents an important marker for drought stress. In the cell, the production and scavenging of ROS and RNS is strictly controlled and the equilibrium can be perturbed by several biotic and abiotic stresses [128]. Plants have evolved complex redox signaling networks in which ROS and RNS are used as signals to regulate normal and stress-related physiological processes including antioxidant mechanisms to combat the toxic effects of ROS and RNS [129,130]. Plants keep ROS under control by an efficient and versatile scavenging system. The antioxidant defense comprises low molecular weight compounds such as GSH, ascorbate (ASC), α-tocopherol, carotenoids, and enzymes including CAT, SOD, and the thiol peroxidases of the PRX and glutathione peroxidase (GPX) type [131].

Thiol peroxidases are linked to the NADPH-thioredoxin reductase (NTR), ferredoxin-dependent TRX reductase (FTR) and GSH/GRX systems [132,133]. Mechanism of ROS production and their scavenging by high antioxidant capacity has been associated with tolerance of plants to abiotic stresses [128]. Recently, a new function was assigned to thiol peroxidases in redox regulation, namely as TRX oxidases [134]. This mechanism allows for reading out the balance between reductive electron input and oxidative electron drainage and tunes the redox and activity state of target proteins.

### 4.1. Effect of Drought Stress on the Antioxidant System and Redox Homeostasis

During drought stress, up-regulation of antioxidant systems occurs at both the transcriptional and post-transcriptional level. Table 4 gives examples for quantitative drought responses of antioxidative enzymes and enzymes involved in regeneration of non-protein antioxidants. APX, catalase (CAT) and GPX represent the principal ROS scavengers in plants. Among these three, APX appears to be induced most strongly on post-transcriptional level (Table 4). In contrast to CAT and GPX, APX is also regulated on transcriptional level based on the data summarized in Table 4. Cytosolic, chloroplastic and peroxisomal APX activities are commonly enhanced in all species of the plant kingdom. The activity of cytosolic APX is increased during drought in pea [135]. The *alx8* mutant (altered expression of APX2) of *Arabidopsis* reveals improved drought tolerance [136,137]. Over-expression of peroxisomal or cytosolic APX from poplar in transgenic tobacco increases plant performance under drought [138,139]. CAT is a tetrameric, heme-containing enzyme that catalyzes the dismutation of H_2_O_2_ into H_2_O and O_2_ in the peroxisome. CAT2 plays a crucial role when the plant is exposed to a severe drought stress [140]. Compared to APX activation, stimulation of CAT is moderate (Table 4). Even though CAT activation seems predominantly taking place on post-transcriptional level, there are examples for complex regulation of CAT activity under severe drought which involves gene expression, translation and protein turnover [141].

Besides APX, other components of the ASC-GSH cycle, namely MDHAR, DHAR, glutathione-S-transferase (GST) and glutathione reductase (GR), work synergistically in different cell compartments. MDHAR, DHAR, GST and GR transcripts and activity are predominantly induced under drought stress (Table 4). Among these four enzymes, GR is activated strongest. GR activation can be compared to the one observed for CAT. In general, upregulation of the ASC-GSH metabolism and associated enzymes efficiently scavenge H_2_O_2_ under drought stress as observed in wheat [167].

Moreover, PRXs are also up-regulated and accumulated in cotton [150], date palm [151] and wheat [161] upon drought (Table 4). This indicates that plants activate compensatory mechanisms to counteract enhanced H_2_O_2_ production in response to drought stress. In addition to their reductive function in detoxifying H_2_O_2_, alkyl hydroperoxide and ONOO^−^, PRX play a role in redox signaling and transmit information on the cell ROS state to target proteins [134,168].

SODs are a class of metalloenzymes that catalyze the dismutation of two molecules of O_2_●^−^ into molecular oxygen and H_2_O_2_. The activation of SOD isoforms (Mn-SOD, Fe-SOD, Cu,Zn-SOD) is interpreted as a measure to counteract O_2_●^−^ accumulation in diverse cell compartments under drought in e.g., *Arabidopsis* [158], blue grass [160], citrus [147], *Coffea canephora* [148], date palm [151], fescue [160], pea [135], poplar [153], tepary bean [145] and wheat [159]. Apparently, SOD is a critical component of the ROS-scavenging system likely by minimizing the reaction of O_2_●^−^ with, e.g., NO to form ONOO^−^, unsaturated fatty acids for peroxidation or with proteins. In line with this assumption transgenic plants overexpressing Cu,Zn-SOD are more tolerant to drought stress [168].

A set of other important proteins belonging to the TRX superfamily is usually highly activated under drought stress. In general, TRXs are induced under different environmental stresses including dehydration, salinity, heat or cold [169]. Under several stresses, atypical and canonical TRX have the capacity to reduce oxidized antioxidant enzymes in the chloroplast, cytosol and mitochondria [170,171]. TRXs are localized in cytosol, chloroplast, mitochondrion, endoplasmic reticulum and nucleus [132]. Strongly responding oxidoreductases are represented by atypical chloroplastic TRX (CDSP32 and CDSP34), cytosolic or mitochondrial NADPH-TRX reductase (NTRA or B), endoplasmic reticulum-associated protein disulfide isomerase (PDI) and canonical cytosolic TRX (TRX h). NTRA-overexpressing plants exhibit extreme drought tolerance with high survival rates, low water loss and reduced ROS accumulation compared to wildtype and *ntra*-knock out plants [144]. However, TRX transcripts and activity measurements in date palm [151] and wheat [161] also indicate a down-regulation of some TRX members in response to drought stress.

### 4.2. Distinct Patterns of Antioxidative Sytem Activation in Sensitive and Tolerant Species

**Figure 3:**
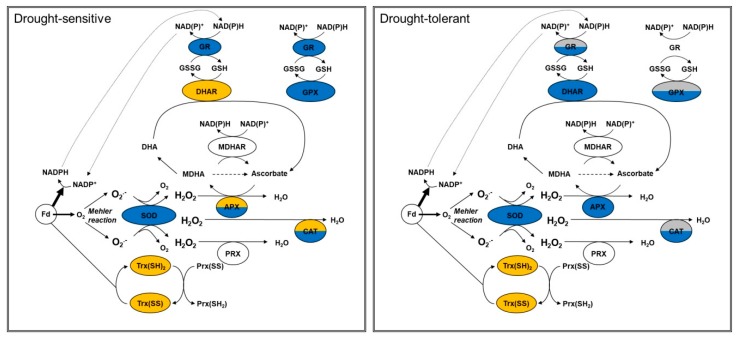
Changes in the activation of the antioxidative system in sensitive and tolerant species. Orange, downregulation, blue, upregulation, grey, no significant changes, no color, no data. APX, ascorbate peroxidase; CAT, catalase; DHAR, dehydroascorbate reductase; Fd, ferredoxin; GPX, glutathione peroxidase; GR, glutathione reductase; MDHAR, monodehydroascorbate reductase; PRX, peroxiredoxin; SOD, superoxide dismutase; TRX, thioredoxin.

As summarized in Figure 2, drought-sensitive species also activate their antioxidative system. The data given in Table 4 confirm this assumption. However, they point out that not only the magnitude of activation might be decisive but also which enzymes are activated. For instance, the activation of the major scavenger APX and CAT is stronger in tolerant species compared to their sensitive counterparts. In contrast, sensitive species activate GPX more than tolerant species. Changes in the activation of the antioxidant system between sensitive and tolerant species are visualized in Figure 3. Obviously, sensitive plants predominantly activate the glutathione-dependent scavenging system, while the ascorbate-dependent system is only induced moderately or are even down-regulated (Figure 3). On the other hand, tolerant species showed a stronger activation of ascorbate-dependent scavenging system compared to the glutathione-dependent system. Moreover, inactivation is only apparent for the TRX-dependent scavenging system in tolerant species. Because drought stress leads to an over-reduction of the electron transport chain, down-regulation of TRX may counteract excessive reduction of target proteins. On the other hand, TRX-dependent reduction of PRX is compromised under this condition. However, PRX can be regenerated by other enzymes like GRX and NTRC [92]. Moreover, drought conditions necessitate a high capacity of detoxifying enzymes such as APX and CAT to suppress ROS accumulation. Furthermore, PRX are involved in redox-signaling [92] which might be their predominant function under drought stress.

There is not much information on drought tolerance and NO signaling. However, a recent study investigated root extracellular and leaf intracellular NO contents in drought-tolerant and –sensitive sugarcane genotypes. Here, drought tolerance was correlated with an increased extracellular NO concentration due to an increased nitrate reductase (NR) activity [89]. Furthermore, the simultaneous decrease in S-nitrosoglutathione reductase (GSNOR) implicates that tolerant plants possess a higher GSNO reservoir. As mentioned before, GSNO is a mobile carrier of NO allowing long distance transport. As observed for roots, likewise, the leaf intracellular NO content was elevated in the tolerant species when compared to the sensitive [89].

When evaluating the role of the ascorbate- and glutathione-dependent pathways in drought tolerance, it must be taken into consideration that the basal levels of the different antioxidants in sensitive and tolerant species were not compared. However, *Arabidopsis* plants lacking the cytosolic APX1 show a collapse in the entire chloroplast-located H_2_O_2_-scavenging system, which is accompanied with increased H_2_O_2_ levels and protein oxidation, respectively [172]. In a direct comparison with TRX-dependent peroxidase activity, APX activity was 7-fold and 2-fold higher in leaf extracts and chloroplasts, respectively [173]. Thus, a predominant role of the ascorbate-dependent antioxidative system should be assumed. At this point, a deeper screen through the literature may not be helpful to test the hypothesis since most studies only present data on changes of selected antioxidant enzymes in a few tolerant and sensitive species. Future investigations should explicitly address the hypothesized role of the ascorbate-dependent ROS defense in drought tolerance in tolerant and sensitive genotypes within plant families. If the hypothesis can be confirmed, the ascorbate-dependent scavenging system can be a target for improving plant tolerance towards drought in biotechnological application.

## 5. The Role of the Antioxidative System in Desiccation Tolerance

Drought stress induces major transcriptional reprogramming in plants via ABA-dependent and ABA-independent pathways regardless whether a plant is sensitive or tolerant to drought. This is also true for resurrection plants. Research has shown that resurrection plants use similar mechanisms and strategies to respond and adapt to drought as sensitive species. However, if processes like perception, signaling and responses are as similar as assumed, which specific features provoke the tolerance to desiccation of vegetative tissues? The major difference to drought-sensitive plants is that the protective machinery of resurrection plants is held in an activated, ‘primed’ state. To achieve this, the basal levels of osmolytes like sugars and polyamines, non-enzymatic and enzymatic antioxidants are often increased in desiccation tolerant plants. High levels of sugars like trehalose, sucrose and raffinose prevent protein denaturation, stabilize membranes and act as ROS scavengers [174,175]. In addition, unique sugars such as the C8-sugar octulose also accumulate to up to 90% of the soluble sugars in photosynthetically active leaves [176]. Despite this, Djilianov and colleagues [177] found that the initial Suc/Fru ratio is increased in the desiccation-tolerant plant *H. rhodopensis* compared to the sensitive species *C. eberhardtii*. The differences and similarities between drought sensitivity, and drought and desiccation tolerance are compiled in Figure 3.

Significant evidence indicates that the strong antioxidant status is a prerequisite of desiccation tolerance in resurrection plants. Thus, glutathione is suggested to be an important player in the dehydration response [178]. The non-enzymatic antioxidants ascorbate and glutathione turn more oxidized during dehydration [177,179], while the total glutathione content increases. The increase in GSSG remains elevated during desiccation of the tolerant species *H. rhodopensis*. In addition, activities of antioxidant enzymes like SOD, peroxidase POD), CAT and GR increase in response to drought in the fern *Selaginella tamariscina* [180]. Resurrection plants are well equipped with genes encoding antioxidant enzymes. For instance, *H. rhodopensis* contains more genes encoding SOD, CAT, MDHAR and GR than the model plant *A. thaliana* [181]. The *H. rhodopensis* genome encodes eight catalase genes and, thus, five more than the *Arabidopsis* genome [181]. Expression of specific *Cat* genes is upregulated following drought/desiccation. The importance of CAT activity during desiccation is shown by an experiment in which leaves were sprayed with the catalase inhibitor 3-aminotriazole (0.1 mmol/L 3-AT). Plants that were treated by 3-AT never recover completely from desiccation and die within a month after the treatment [181]. The increased sensitivity of dehydrating plants to CAT inhibitors is interpreted as indication of enhanced photorespiration due to stomatal closure, lack of intercellular CO_2_, enhanced oxygenation of RUBISCO and therefore stimulated release of H_2_O_2_ by glycolate oxidase in the peroxisome. CAT is needed to detoxify the released H_2_O_2_ and therefore inhibited CAT disturbs redox and ROS homeostasis under drought.

Wang and colleagues [180] compiled drought/dehydration-responsive proteins from both resurrection and common plants [180]. The comparison of tolerant with sensitive phenotypes highlights the role of the antioxidant system in drought tolerance. For instance, CAT, APX and SOD levels are up-regulated in the drought-tolerant CE704 genotype (maize), while CAT and APX levels decreased in the drought-sensitive genotype 2023 [182]. In wheat, TRX-h and glutathione S-transferase are selectively upregulated in the drought-tolerant genotype Khazar-1 [161].

It should be noted that dehydration tolerance depends on additional features of the plants apart from adjusting metabolism including the antioxidant system. Massive water loss usually causes mechanical disruption in hygrophytic and mesophytic plants, e.g., the rupture of the tonoplast/plasmamembrane/cell wall junctions. Such irreversible mechanical damage is prevented in resurrection plants such as *Craterostigma plantagineum* where the tissue shrinks proportionally to the water loss. Thus, special anatomical properties like leaf curling and structurally flexible vessels are important features of dehydration tolerance [183,184].

## 6. Conclusion and Perspective

Drought tolerance depends on conditional activation of the acclimation program during initial phases of water loss. This also applies for thallophytic and cormophytic resurrection plants which need a hardening period for full expression of the tolerance trait [183,185]. As pointed out in this review, different drought stress regimes and time points of analysis result in distinct states of the ROS and RNS network and the antioxidant defense system. In the initial phases of dehydration, the activation of the hardening program decisively involves the generation of ROS and RNS which assist in activating the redox regulatory network and appropriate gene expression and protein accumulation. It was out of focus of this review to describe the intimate link between ROS, RNS and hormone signaling like salicylic acid and abscisic acid [186]. In the end ROS and RNS define a regulatory framework of the cell and contribute to link the stress impact to gene expression and whole plant performance [187].

At present our knowledge on specific subcellular ROS, RNS and redox patterns still falls short of the requirements for understanding the drought acclimation response in its entirety. Cell imaging with roGFP for glutathione redox state [188] and Hyper for H_2_O_2_ [189] will provide important insight on subcellular responses. In addition, in depth redox proteomics detecting the redox state of also low abundant proteins will provide a global view with subcellular resolution.

There is a need to assess the various PTMs in the proteome simultaneously. This is a challenge for current proteomics which for technical reasons often focuses on single or few PTMs only [190]. As functional readout of ROS and RNS, such approaches will realize the necessary temporal and spatial resolution since ROS and RNS partly antagonize each other. Nevertheless, the presence of both reactive species is necessary for full drought acclimation. Additionally, the reaction of NO with O_2_●^−^ generates the highly reactive ONOO^−^ which directly nitrates proteins. Cysteine oxidation and tyrosine nitrations are PTMs that change the activity of its target enzymes. Proteomics may tackle this challenge.

Along with the activation of the antioxidative system, other stress markers often increase during periods of progressive dehydration, e.g., H_2_O_2_ as indicator of redox imbalance, MDA as lipid oxidation product, glyoxylate linked to photorespiration, glutathione as antioxidant, glutamate and proline as precursor and compatible solute, and zeaxanthin with its role in photoprotection. The consensus of what defines drought tolerance is that many traits are needed to prevent biochemical or physiological impairment during water deficit. Several traits contribute to drought tolerance and include reduced water loss, build-up of osmotic potential, synthesis of compatible solutes, dissipation of excess energy, activation of antioxidant defense and repair systems, generation of sclerenchymatic tissue, strengthening the plasmamembrane-cell wall interaction and other mechanisms of growth adjustment such as differentiation of smaller leaves. The recovery from water depletion is affected by light intensity with often negative interference, i.e., slower recovery at high light.

Taken together, strategies to improve drought tolerance in crops need to target several metabolic pathways at the same time. Certainly, the activation of the antioxidative system following drought is one important goal. Attention should also be drawn to the pathways that are selected to increase drought tolerance. In the first instance, overexpressing of certain enzymes can lead to a beneficial increase in drought tolerance, but may delay germination and development for months and, thus, interfere with the growing season. Thus, biotechnological approaches should take into account the temporal and spatial signaling aspect in drought stress acclimation.

## Figures and Tables

**Table 1:** Classification of drought stress by different units that describe the water availability for different species at the various stages of drought stress.

Plant Species	Unit	Control	Mild	Moderate	Severe	Very Severe	Length of Stress Application	Reference
*Camellia sinensis* (Tea)	Soil moisture content [%]	19.5	15.2	10.17	5.54		week(s)	[21]
*Arabidopsis thaliana*	Water content [g water/g dry soil]	2.2	1.2		0.7		weeks	[22]
*Arabidopsis thaliana*	Relative soil water content [%]	85–90		45–50	30–35		week(s)	[23]
*Biserrula pelecinus*	Water holding capacity	70–90	40–60		20–40		month	[24]
Common bean	Soil field capacity [%]	90	70		50	30	weeks	[17]
Jujube tree	Relative soil moisture [%]	80	70	60	40			[25]
Lemon balm and thyme	Relative soil water content [%]	70		40	25		months	[26]
*Malus hupehensis*	Soil field capacity [%]	75–85		45–55			months	[27]
Poplar	Relative soil water content [%]	70	45		20		month	[28]
Soybean	g_s_ intervals [mol H_2_O m^−2^s^−1^]	>0.2	0.1–0.2		<0.1		week	[29]
Tomato	Soil field capacity [%]	100		50			weeks	[30]
*Valeriana officinales*	Available water content/relative water content (%)	100/77.3	70/67.2		65.1/50	51.4/30	months	[31]
Wheat	Relative soil water content [%]	80–90	35–43		20–25		week	[32]
Wheat	Relative water content [%]	80–100	60–80		40–60		weeks	[33]
Wheat	Soil field capacity [%]	85		55			months	[34]
*Populus deltoides*	Water potential [MPa]	−0.1	−0.5		−1.26		week	[35]
Wheat and maize	Water potential [MPa] in the presence of PEG6000		−0.4	−0.8	−1.5		week	[36]

g: gram(s).

**Table 2:** Exemplary experimental design for testing drought tolerance in different plant species.

Plant Species	Drought Stress (Age of Plants, Duration, Re-Watering)	Medium	Reference
*Arabidopsis thaliana*	2-weeks-old, 13 d no water, re-hydration for 2 d	soil	[37]
*Arabidopsis thaliana*	2-weeks-old, 5 d no water	MS medium	[38]
*Arabidopsis thaliana*	2-weeks-old, 12 d no water, re-hydration for 4 days	soil	[39]
Rice	2-weeks-old, 4 d 20% PEG-6000, 1–10 d re-watering	hydroponics	[40]
Rice	40-days-old, 7 d no water, 1–10 d re-watering	soil	[40]
Sugarcane	120-days-old, 10 d no water, re-watering	soil	[41]
Tobacco	14 d without water, 3 d re-watering	soil	[42]
Tomato	8-weeks old, up to 21 d no water	soil	[43]
Wheat	3-leaves stage, 72 h 20% PEG-6000 in 1/2 Hoagland solution (HS), 1 d re-watering with 1/2 HS	hydroponics	[44]

d, day(s); h, hour(s); MS medium, Murashige–Skoog medium; PEG, polyethylene glycol.

**Table 3:** Changes in reactive oxygen species (ROS) and nitric oxide (NO) amounts upon drought or osmotic stress treatment in various plant species. Data originate from green leaf tissue if not indicated otherwise. Increase in percent was chosen due to different detection methods with different units. Effects were estimated from graphs, figures and tables if not directly given in the text or supplements.

ROS/RNS Species	Plant Species	Stress Application	Observed Change in ROS/RNS Concentration (% Relative to Control)	Reference
H_2_O_2_	*Ailanthus altissima*	No water for 14 d	+166	[63]
	*Arabidopsis thaliana*	200 mmol/L mannitol for 6 h	+50	[64]
	*Brassica rapus*	10% PEG for 2 d 20% PEG for 2 d	+30 +65	[65]
	*Citrus reticulata*	No water for 3 d No water for 6 d No water for 9 d	+16,6 +37,5 +45,5	[66]
	*Cleome spinosa*	No water for 10 d	+25	[67]
	*Crambe abyssinica*	50% MWHC for 32 h 50% MWHC for 136 h	+15 +84	[68]
	*Helianthus annuus* cultivars	40% SFC for 21 d	Variable, see literature	[69]
	*Helianthus annuus* Aydin *Helianthus annuus* Musala	10% PEG for 5 d 20% PEG for 5 d 10% PEG for 5 d 20% PEG for 5 d	+68 +50 +15 +30	[70]
	*Medicago sativa*	No water for 7 d	+490	[71]
	*Oryza sativa*	200 mmol/L mannitol for 2 d	+200	[61]
	*Oryza sativa* callus	5% PEG for 28 d 10% PEG for 28 d 15% PEG for 28 d 20% PEG for 28 d	+200 +225 +300 +380	[62]
	*Oryza sativa* roots and leaves	−0.5 MPa for 1 d −2.0 MPa for 1 d	Age dependent, see literature	[72]
	*Sorghumbicolor* M-81E *Sorghum bicolor* Roma	No water for 7 d No water for 7 d	+28.9 +54.9	[73]
	*Stevia rebaudiana*	15% PEG for 30 d	+220	[74]
	*Triticum aestivum*	50% RWC for 12 d	+40	[75]
	*Triticum aestivum* seedlings	15% PEG for 2 d 15% PEG for 4 d 15% PEG for 6 d	+45 +200 +280	[76]
	*Triticum aestivum* (booting) *Triticum aestivum* (filling)	No water for 52 d No water for 69 d	+70 +43	[77]
	*Zea mays* growth zones	20 % less SWC till 3 d after 5th leaf	Doubled across all zones	[60]
O_2_●^−^	*Crambe abyssinica*	50% MWHC for 32 h	+18	[68]
	*Helianthus annuus*	10 % PEG for 1 d	−60	[78]
	*Oryza sativa* roots and leaves	−0.5 MPa for 1 d −2.0 MPa for 1 d	Age dependent, see literature	[72]
	*Sorghum bicolor*	10% PEG for 1 d	−22.5	[78]
NO	*Ailanthus altissima*	No water for 14 d	+125	[63]
	*Ananas comosus*	30% PEG for 25 d	Variable emission, see literature	[79]
	*Arabidopsis thaliana*	No water for 4 d	+150	[80]
	*Citrus aurantium*	13% PEG for 12 d	+150	[81]
	*Cucumis sativus*	Root aeration for 5, 10, 15 h plus rewatering	Variable, see literature	[82]
	*Hordeum vulgare*	No water for 18 d	Doubled production rate	[83]
	*Lotus japonicus* roots and leaves	No water for 5 d	+80 +33	[84]
	*Medicago truncatula* roots and leaves	No water for 3, 9, 11 d plus rewatering	Variable, see literature	[85]
	*Oryza sativa*	200 mmol/L mannitol for 1, 6, 24 h	Variable, see literature	[61]
	*Oryza sativa*	No water for 9 d	+200	[86]
	*Oryza sativa* seeds	20% PEG for 1d	−75	[87]
	*Poncirus trifoliate*	No water 6 h	+200	[88]
	*Saccharum spp.* roots and leaves	−0.4 MPa (PEG) for 1 d	Variable, see literature	[89]

MWHC, maximum water holding capacity; RWC, relative water content; SWC, soil water content; SFC, soil field capacity; d, day(s); h, hour(s.).

**Table 4:** Antioxidant enzymes regulated in plants under drought.

Antioxidative Enzyme	Plant Species	Transcriptional Regulation	Post-Transcriptional Regulation	Reference
Ascorbate peroxidase (APX)	Alfalfa		only severe: 15%	[142]
	*Arabidopsis thaliana*	APX1 1.66-fold	800%	[143]
	*Arabidopsis thaliana*	APX1 ns		[144]
	*Arabidopsis thaliana*	APX3 2-fold		[143]
	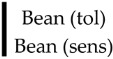		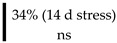	[145] [145]
	*Carrizo citrange *	APX2 5.5-fold	50%	[146]
	*Carrizo citrange *	cAPX 2-fold	Total ns	[147]
	*Cleopatra mandarin*	APX2 10-fold	50%	[146]
	*Cleopatra mandarin*	cAPX 0.5-fold	Total ns	[147]
	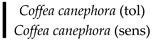		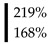	[148] [148]
	Cotton (tol)		up to 50%	[149]
	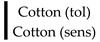		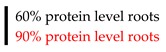	[150] [150]
	Date Palm	APX-46 4-fold		[151]
	Date Palm	APX-1 4-fold		[151]
	Maize		25%	[152]
	Pea	cAPX1 3-fold (not log2-fold)	cAPX1 50%	[136]
	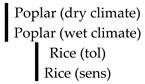		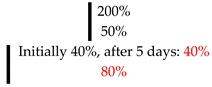	[153] [153] [154] [154]
	Tobacco	APXI 299 %	300%	[155]
	Tobacco	thyAPX and strAPX ns		[156]
	Wheat	2.29-fold (rel. expression)	35%	[157]
Catalase (CAT)	Alfalfa		100 % (moderate)	[142]
	Alfalfa		ns (severe)	[142]
	*Arabidopsis thaliana*		30%	[158]
	*Carrizo citrange *	1.5-fold	ns	[147]
	*Cleopatra mandarin *	1.5-fold	ns	[147]
	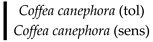		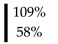	[148] [148]
	Maize		50%	[152]
	Pea		100%	[136]
	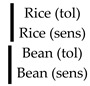		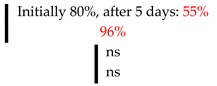	[154] [154] [145] [145]
	Tobacco	CAT1-2 ns		[156]
	Tobacco	CAT3 2.4-fold (rel. expression)	45%	[155]
	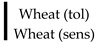		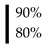	[159] [159]
	Cotton		up to 50%	[149]
	Fescue		33%	[160]
Dehydroascorbate reductase (DHAR)	Date Palm	DHAR-25 1.4 fold		[151]
	Date Palm	DHAR-2 1.4-fold		[151]
	Wheat	2.3-fold (rel. expression)	44%	[157]
	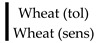		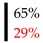	[161] [161]
Glutathione peroxidase (GPX)	Alfalfa		ns	[142]
	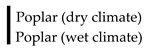		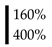	[153] [153]
	Potato	2.9-fold (rel. expression)		[162]
	Tortula		50%	[163]
	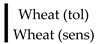		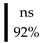	[164] [164]
Glutathione reductase (GR)	*Arabidopsis thaliana*		65%	[158]
	*Carrizo citrange*	2-fold	90%	[147]
	*Cleopatra mandarin*	2-fold	50%	[147]
	Cotton		up to 80%	[149]
	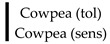	3.5-fold (rel. expression) 4-fold (rel. expression)	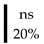	[165] [165]
	Maize		33%	[152]
	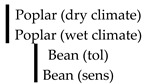		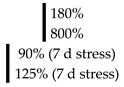	[153] [153] [145] [145]
	Tobacco	1.6-fold (rel. expression)		[156]
	Tobacco		35%	[155]
	Tortula		100%	[163]
	Wheat	2.1-fold (rel. expression)	30%	[157]
	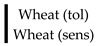		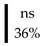	[164] [164]
Glutathione *S*-transferase (GST)	Tortula		40%	[163]
	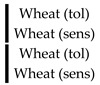		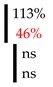	[161] [161] [164] [164]
Monodehydroascorbate reductase (MDHAR)	Wheat	2.3-fold (rel. expression)	65%	[157]
	Tobacco	1.6-fold (rel. expression)		[156]
Protein disulphide isomerase (PDI)	Stiff brome	BdPDIL1-1 > 1-fold (rel. expression)		[166]
	Stiff brome	BdPDIL1-2 0.67-fold,		[166]
	Stiff brome	BdPDIL7-2 0.33-fold		[166]
	Stiff brome	BdPDIL2-1 > 1-fold (rel. expression)		[166]
	Stiff brome	BdPDIL3-1, BdPDIL5-1 and BdPDIL8-1 (between 0.33 and 1-fold)		[166]
Peroxiredoxin (PRX)	Date Palm	PRXR-18 1.1-fold		[151]
	Date Palm	PRXR-1 1.5.fold		[151]
	Date Palm	PRXR-2 4.3-fold		[151]
Superoxide dismutase (SOD)	Alfalfa		Total SOD ns	[142]
	Alfalfa		MnSOD 30%	[142]
	*Arabidopsis thaliana*		100%	[158]
	*Arabidopsis thaliana*	ns		[144]
	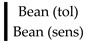		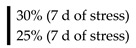	[145] [145]
	*Carrizo citrange *	CuZnSOD 2-fold	ns	[147]
	*Cleopatra mandarin*	FeSOD 1.5-fold	100%	[147]
	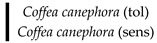		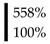	[148] [148]
	Date Palm		up to 450%	[151]
	Blue Grass		100% (25 d of stress)	[160]
	Fescue		30% (25 d of stress)	[160]
	Maize	SOD-13 1.2-fold	20%	[152]
	Maize	SOD-11 1.26-fold		[152]
	Pea		100% (chloroplast and cytosol)	[136]
	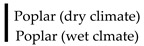	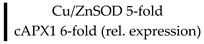		[153] [153]
	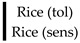		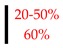	[154] [154]
	Tobacco	ns	ns	[155]
	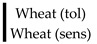		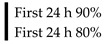	[159] [159]
Thioredoxin (TRX)	Date Palm	TRX-40 1.1-fold TRX-44 1.3-fold TRX -37 1.3-fold TRX -16 1.3-fold TRX -31 1.3-fold TRX -12 1.1-fold		[151]
	Date Palm			[151]
	Date Palm			[151]
	Date Palm			[151]
	Date Palm			[151]
	Date Palm			[151]
	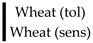		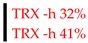	[161] [161]

APX, ascorbate peroxidase; CAT, catalase; DHAR, dehydroascorbate reductase; GPX, glutathione peroxidase; GR, glutathione reductase; GST, glutathione-S transferase; MDHAR, monodehydroascorbate reductase; PDI, protein disulfide isomerase; PRX, peroxiredoxin; SOD, superoxide dismutase; TRX, thioredoxin. Black color, up-regulation; red color, down-regulation; ns, not significantly changed.

## Abbreviations

ABA	abscisic acid;
AOX	alternative oxidase;
APX	ascorbate peroxidase;
ASC	ascorbate;
CAM	crassulacean acid metabolism,
CAT	catalase;
CO_2_	carbon dioxide;
cys	cysteine;
d	day(s);
DAF	diaminofluorescein;
DAR	diaminorhodamine;
DHAR	dehydroascorbate reductase;
ELIP	early light inducible protein;
Suc/Fru	sucrose to fructose ratio;
ETC	electron transport chain;
FTR	ferredoxin-dependent TRX reductase;
g	grams;
GPX	glutathione peroxidase;
GPX	glutathione peroxidase;
GR	glutathione reductase;
GRX	glutaredoxin;
GSH	glutathione;
GSNO	nitrosoglutathione;
GSNOR	S-nitrosoglutathione reductase;
GST	glutathione-S transferase;
h	hours;
H_2_O	water;
H_2_O_2_	hydrogen peroxide;
HS	Hoagland solution;
HSP	heat shock protein;
LEA	late embryogenesis abundant protein;
MDA	malondialdehyde;
MDHAR	monodehydroascorbate reductase;
Met	methionine;
MPa	megapascal;
MWHC	maximum water holding capacity;
NAD+/NADH	oxidized/reduced nicotinamide adenine dinucleotide;
NADP+/NADPH	oxidized/reduced nicotinamide adenine dinucleotide phosphate;
NO	nitric oxide;
NR	nitrate reductase;
NTR	NADPH–thioredoxin reductase;
NTRC	NADPH-dependent thioredoxin reductase C;
O_2_	molecular oxygen;
O_2_●^−^	superoxide anion;
ONOO^−^	peroxynitrite;
PDI	protein disulfide isomerase;
PEG	polyethylene glycol;
PTM	posttranslational modification;
PRX	peroxiredoxin;
PSI	photosystem I;
PUCPs	plant uncoupling proteins;
RBOH	respiratory burst oxidase homolog;
RLK	receptor-like kinase;
RNS	reactive nitrogen species;
ROS	reactive oxygen species;
RWC	relative water content;
SFC	soil field capacity;
SOD	superoxide dismutase;
SWC	soil water content;
TRX	thioredoxin;
WAKs	cell wall-associated kinases
